# Physicochemical Characterization and Polyphenolic Content of Beninese Honeys

**DOI:** 10.1155/2017/6571089

**Published:** 2017-09-07

**Authors:** Sênan Christa Lokossou, Fidèle Paul Tchobo, Hounnankpon Yédomonhan, Mohamed Mansourou Soumanou

**Affiliations:** ^1^Unit of Food and Enzymatic Engineering Research, Polytechnic School of Abomey-Calavi, University of Abomey-Calavi, 01 BP 2009 Cotonou, Benin; ^2^Laboratory of Botany and Plant Ecology, Faculty of Sciences and Technologies, University of Abomey-Calavi, 01 BP 4521 Cotonou, Benin

## Abstract

The physicochemical and phytochemical analyses of honeys (*n* = 50) from Sudanese, Sudano-Guinean, and Guinean areas of Benin were investigated. Results showed that dark amber is the dominant color. Moisture content ranged from 15.50% to 23.50%, and 72% of honeys respected the Codex Alimentarius recommendation. pH varied between 2.87 and 6.15, and free acidity ranged from 9.00 to 39.00 meq/kg. Electrical conductivity varied from 0.37 to 1.43 mS/cm. The content in fructose varied from 21.67% to 94.21%, and proline content ranged between 306.31 and 1187.93 mg/kg. All physicochemical characteristics varied with the areas. A negative correlation was found between pH and moisture content (*r* = −0.55; *p* < 0.01). A positive correlation was established between pH and conductivity (*r* = 0.79; *p* < 0.01) and between proline and color (*r* = 0.44; *p* < 0.01). Total phenolic content varied between 55.97 and 224.99 mg GAE/100 g, and flavonoid content ranged between 1.43 and 29.81 mg CAE/100 g. Flavonoid was positively correlated with color (*r* = 0.78; *p* < 0.01) and proline (*r* = 0.47; *p* < 0.01). Tukey's test revealed differences between total phenolic and flavonoid contents of honeys from five areas (*p* < 0.01). In general, Sudanese and Sudano-Guinean honeys showed potential toward therapeutic applications because of their high phenolic contents.

## 1. Introduction

Honey is a natural sweet substance produced by honeybees,* Apis mellifera *(Linnaeus, 1758), from the nectar of plants (blossoms), the secretions of living parts of plants, or excretions of plant-sucking insects present on the living parts of plants. The bees collect, transform (by combining them with specific substances of their own), deposit, dehydrate, store, and leave the collected nectar in the honeycomb to ripen and mature [1]. Honey has various nutritional, medicinal, and prophylactic properties provided by its various chemical constituents [2]. Honey has been reported to contain about 200 substances (complex mixture of sugars, but also small amounts of other constituents such as minerals, proteins, vitamins, organic acids, flavonoids, phenolic acids, enzymes, and other phytochemicals) and is considered as an important component of traditional medicine [3]. Its composition is rather variable and depends primarily on its floral origin; however, certain external factors, such as seasonal and environmental factors, as well as processing methods, also play a role [2, 4–8]. Besides, the quality of honey is mainly determined by its sensorial, chemical, physical, and microbiological characteristics. The major physicochemical quality criteria are moisture content, electrical conductivity, ash content, reducing and nonreducing sugar content, free acidity, diastase activity, and hydroxymethylfurfural content [9].

Elsewhere, it has been demonstrated that some of the constituents present in honey have antioxidant properties. These include phenolic acids and flavonoids [10], certain enzymes (glucose oxidase and catalase) [11], ascorbic acid, proteins, and carotenoids [6]. The botanical origin of honey influences its antioxidant activity, while processing, handling, and storage only affect this activity to a minor degree. Several studies have shown that antioxidant activity is strongly correlated with the total phenolic content [12–15].

Benin is subdivided into three phytogeographical areas, each characterized by specific flora [16]. As studies clearly indicate the variation in honey quality with distinct floral origins, this study was undertaken to compare the physicochemical characteristics, as well as total phenolic and flavonoid contents of honeys obtained from different phytogeographical areas of Benin.

## 2. Material and Methods

**Figure 1:**
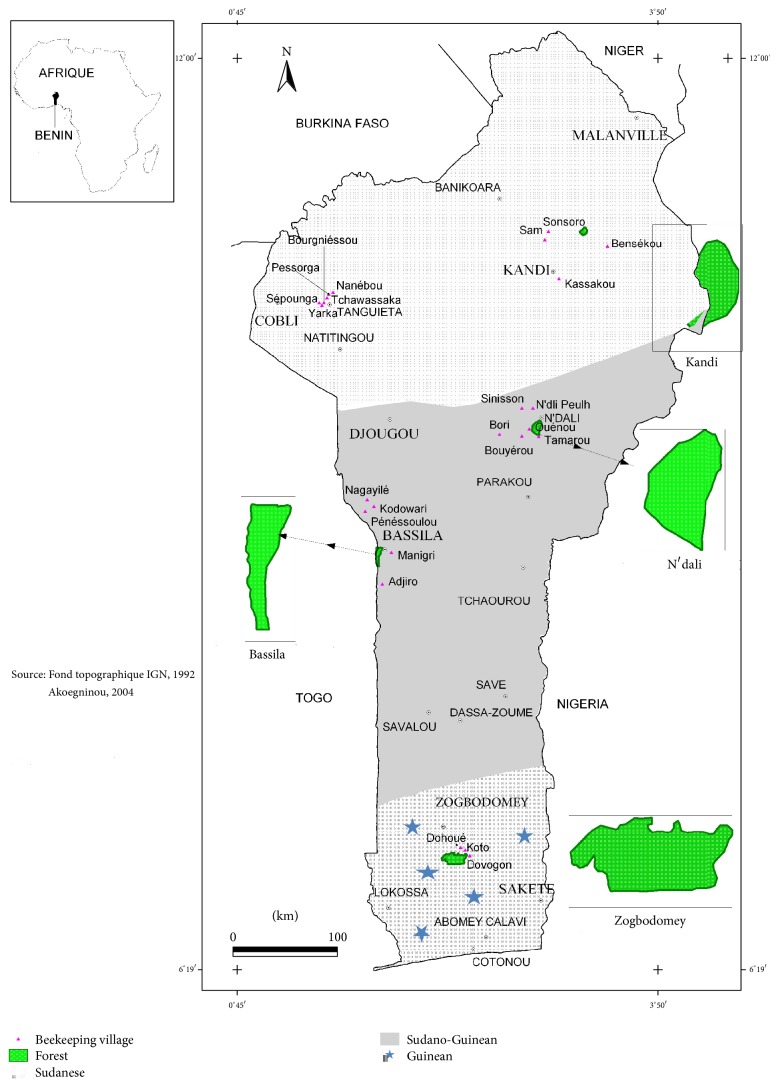
Geographical localization of the towns.

Benin is subdivided into three phytogeographical areas (Sudanese, Sudano-Guinean, and Guinean). Preliminary investigations showed that Bassila (zone SG1) and N'Dali (zone SG2) in the Sudano-Guinean area, Tanguieta (zone S1) and Kandi in the Sudanese area, and Zogbodomey (zone G) in the Guinean area represent five towns where beekeeping is practiced and high quantities of honey are produced (Figure 1). Fifty samples were collected from these five towns: ten samples per zone. Each phytogeographical area is characterized by its flora [16]. Honeys were collected between March and December 2016 in agreement with honey harvest periods. All samples were kept at 4°C.

### 2.1. Physicochemical Properties

Moisture content, pH, acidity, and electrical conductivity were determined by standard methods defined by the International Honey Commission [17]. Moisture content was determined using a honey hand refractometer (Honey Tester 68–92% N°600048 meopta), pH and free acidity were determined by titration to pH 8.3, and electrical conductivity was determined using a HANNA DIST2 hand conductometer. Color was determined according to the White [18] method, based on the Pfund indicator calculation. Absorbance was measured at 635 nm using 50% filtered honey solutions and the Pfund indicator calculated as −38.70 + 371.39 *∗* Absorbance.

Sugar content was evaluated through the phenol-sulfuric method developed by Dubois et al. [19]. For each honey solution (1 mL), 0.5 mL of phenol solution (5% in water) was added, followed by the addition of 2.5 mL of sulfuric acid. The mixture was then shaken and immediately stored at 25–30°C for twenty minutes. Absorbance was subsequently read at 485 nm. Fructose content, expressed in grams per 100 grams, was calculated using a calibration curve prepared from a fructose standard solution, that was analyzed in the same way as the honey samples.

Proline content was determined through the International Honey Commission method [17]. Each honey solution (0.5 mL) was mixed with 1 mL of formic acid (80%) and 1 mL of ninhydrin solution (3% in the ethanol). The mixture was then shaken vigorously for fifteen minutes and placed in a bath containing boiling water for an additional fifteen minutes. Then, the mixture was transferred into a water bath set at 70°C for ten minutes. Subsequently, 5 mL of 50% 2-propanol solution was added to the mixture, which was then left to cool down. The absorbance was subsequently read at 520 nm. Proline content, expressed in milligrams per kilogram, was calculated using a calibration curve prepared with a proline standard solution, that was analyzed in the same way as the honey samples.

The Folin-Ciocalteu method was used to determine the total phenolic content, as reported by Singleton et al. [20]. Each honey solution was filtered (0.5 mL) and then mixed with 2.5 mL of 0.2 N Folin-Ciocalteu reagent for five minutes, before adding 2 mL of sodium carbonate. After incubation in the dark for two hours, the absorbance was read at 760 nm. Gallic acid was used to generate the calibration curve. The total phenolic content (TPC) was expressed in milligrams of gallic acid equivalents (GAE) per 100 g of honey.

Flavonoid content was determined using the colorimetric method described by Ita [21]. Each honey solution (1 mL) was mixed with 0.3 mL of sodium nitrite solution. After five minutes, 0.5 mL of aluminum chloride was added. The mixture was then homogenized and left to react for six minutes. Two milliliters of potassium hydroxide (KOH, 1 M) solution was subsequently added, and absorbance was read at 510 nm. Catechin was used to produce the calibration curve. Total flavonoid content (TFC) was expressed in milligrams of catechin equivalent (CAE) per 100 g of honey.

All analyses were carried out in duplicate. Statistical analysis was performed using IBM SPSS (IBM corporation, Java) and Minitab 14 Release (statistical software). Data were subjected to a one-way variance analysis (ANOVA) for mean comparison, and significant interhoneys differences were estimated using the Tukey multiple-range test. The results are expressed as mean ± standard deviations (SD). Correlations were calculated according to Pearson's test. Differences at *p* ≤ 0.05 were considered as statistically significant. The dendrogram was made with Minitab based on Ward algorithm and Euclidean distance.

## 3. Results and Discussion

The physicochemical results obtained for the honeys studied are shown as arithmetical means plus standard deviations in Table 1.

Honey color is the first physical property perceived by the consumer. In this study, honey color varies from 105.03 ± 1.05 mm Pfund (zone S1) to 752.36.94 ± 1.05 mm Pfund (zone SG2). Based on the NC 371-04 [22] classification, samples are classified as either amber honeys (6%) (Pfund value between 86 and 114) or dark amber honeys (94%) (Pfund values higher than 114). Honeys from the Sudano-Guinean (N'dali) and Sudanese (Kandi) zones are the darkest. Significant differences are observed between the means obtained from different zones (*p* < 0.01). Delphine and Joseph [23] observed that dark amber is the dominant color for honeys produced in Cameroon. White [4] reported that it is an important parameter to consider when assessing the quality and market value of honey. Lighter honeys are marketed for direct consumption while darker ones, which contain more minerals, have higher market values. According to Reshma et al. [24], the color of honey is closely related to its chemical composition and more particularly, to the presence of pigments such as chlorophylls, carotenoids, flavonoids, and derivatives of tannins and polyphenols. Honey color reflects the melliferous flora harvested by bees and varies according to the seasons, the harvesting technics, and the treatment of honey [23]. Many researchers found that honeys with dark color have higher TPC and, consequently, higher antioxidant properties [13, 14].

Moisture content is an important quality parameter to take into consideration when evaluating honey quality. The moisture content values obtained ranged from 15.5%  ±  0.00% (zone SG1) to 23.50%  ±  0.00% (zone G). Honeys used in this studies have higher moisture contents than Algeria, Cameroon, and Palestine honeys, whose values range from 13% to 21.42% [23, 25, 26]. However, our results are still similar to those obtained (18–23.6%) from Indian multifloral honeys [24]. These honeys have less moisture content than Nigerian ones (18.30–30.30%) [27]. Indeed, both the Codex Alimentarius [1] and European Union [9] recommend a humidity value ≤ 20%. High moisture could increase honey fermentation. Sudanese zone samples showed the lowest values (Kandi), whereas the highest values were observed in Guinean honeys. Therefore, Guinean honeys do not meet the requirements and will rapidly ferment.

pH values varied between 2.87 ± 0.01 (zone G) and 6.15 ± 0.07 (zone SG1), and free acidity varied from 9.00 ± 0.00 (zone SG1) to 39.00 ± 0.00 (zone G) meq/kg. None of the samples exceeded the limit (≤50 meq/kg) considered as the freshness index for all honeys, indicating the absence of any unwanted fermentation [28]. The free acidity values obtained are inferior to those found by Reshma et al. [24] and Meda et al. [29] in India and Burkina Faso, respectively. Similar values were obtained by Achour and Khali [25], as well as Sodré et al. [30], in Algerian and Brazilian honeys, respectively. Guinean zone samples are more acidic than Sudano-Guinean samples. The higher acidity values of Guinean zone honeys imply their good conservation, as it creates an inappropriate environment for bacterial growth. The acidity variation among honey samples is dependent on floral types [31]. However, according to Alvarez-Suarez et al. [6], honey is naturally acidic, regardless of its geographical origin. A negative correlation was found between pH and moisture content (*r* = −0.55; *p* < 0.01). Therefore, honeys with high moisture contents also display low pH. This relation contributes to the good conservation of moist honeys.

Electrical conductivity (EC) of honey is proportional and shows a linear relation, with ashes content. According to Estevinho et al. [32], while the ashes content reflects the quantity of inorganic residues after carbonization, the conductivity reflects the amount of organic and inorganic ionizable substances. The EC values of honeys varied between 0.37 ± 0.00 (zone G) and 1.43 ± 0.00 mS/cm (Zone 1). Means are significantly different (*p* < 0.01) and indicate high EC of Sudano-Guinean honeys (Bassila) and low values for Guinean samples. These results are in the same range (0.24–1.57 mS/cm) than those found by Adenekan et al. [33] and Mekious et al. [34]. Additionally, there is a correlation between pH and electrical conductivity (*r* = 0.79; *p* < 0.01).

Sugar content, expressed in percentage of fructose (*y* = 0.0051*x* − 0.00009; *R*^2^ = 0.999), varied from 21.67%  ±  0.06% to 94.21%  ±  0.12%. Sugar content varied considerably according to zones (*p* < 0.01), but the samples obtained from the Sudano-Guinean (N'Dali) and Guinean zones showed particularly high qualities in terms of sugar content. Proline contents (*y* = 0.0241*x* + 0.0005; *R*^2^ = 0.999) varied from 306.31 ± 0.84 to 1187.93 ± 3.16 mg/kg. Proline is considered as the main amino acid in honey, and its value must be superior to 180 mg/kg. The amount of proline is an indicator of purity, and its level decreases significantly in soiled honeys. Content of proline varied significantly among zones (*p* < 0.01). The higher values were obtained from Sudanese (Kandi) and Sudano-Guinean (N'Dali) samples. Values of proline content measured in this study are higher than those obtained from Turkish honeys [35] but range between 169 and 2169 mg/kg obtained by Meda et al. [10] and Djossou et al. [36]. The level of proline has been reported to vary according to the honey flora [37]. Positive correlations were found between proline and color (*r* = 0.44; *p* < 0.01).

Polyphenols are molecules displaying antioxidant properties. These molecules are paramount toward conferring medicinal properties to honeys. Various floral honeys with high polyphenol contents are consumed as medicinal products [35]. Antioxidants, for example, play an important role in food preservation and in human health, by combating damage caused by oxidizing agents such as oxygen.

**Figure 2:**
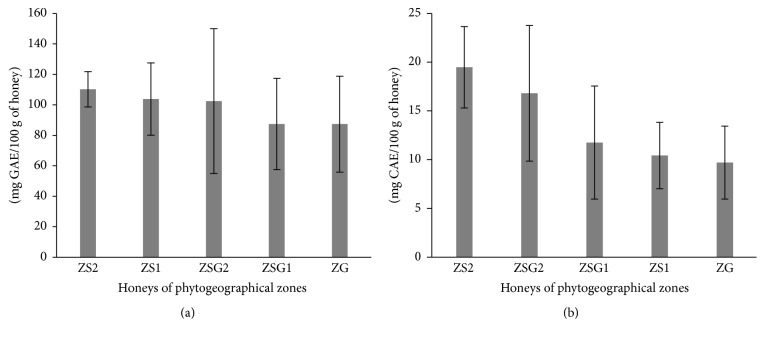
(a) Means of total phenolic content and (b) means of flavonoids content.

TPC of honeys varied between 55.97 ± 0.00 and 224.99 ± 0.78 mg GAE/100 g of honey. Honeys from the Sudanese zone have higher TPC values than honeys from the Guinean and Sudano-Guinean zones (Figure 2(a)). The TPC values measured in this study are higher than those obtained (16.02–120 mg/100 g) by Reshma et al. [24], Can et al. [35], and Buba et al. [38] but are similar to the results reported by Djossou et al. [36] and Ouchemoukh et al. [39]. TFC of honeys (*y* = 10.105*x*  −  0.1569; *R*^2^ = 0.9989) ranged between 1.43 ± 0.00 and 29.81 ± 0.07 mg CAE/100 g of honey. Beninese honeys contain higher flavonoid levels than Cuban (1.09–2.52 mg QE/100 g) and Bangladeshi (1.15–11.67 CAE/100 g) ones [6, 40]. Honeys from the Guinean area show the lowest values of flavonoid content (Figure 2(b)). Tukey's test revealed differences between phenolic and flavonoid contents across the five zones (*p* < 0.01). Correlations were found between flavonoid and proline contents (*r* = 0.47; *p* < 0.01) and between flavonoid content and color (*r* = 0.78; *p* < 0.01). The darker honeys display the highest antioxidant properties and the highest phenolic, flavone, and flavonol contents [41].

**Figure 3:**
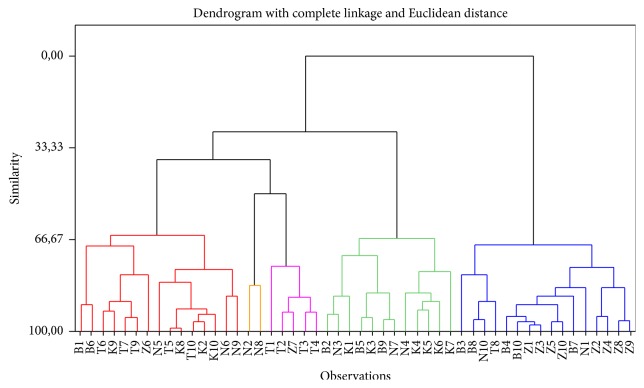
Classification of honeys.

For 50% similarity, the dendrogram shows 5 clusters (Figure 3), whose characteristics are presented in Table 2. This classification shows that most of the Sudanese area honeys do not have any dominant characteristic. These samples are very scattered across the clusters. In general, Sudanese zone honeys have high total phenolic and flavonoid contents (Clusters 2 and 5). Guinean honeys are clearly characterized by low proline and polyphenolic contents (Cluster 3). Sudano-Guinean samples show high contents in proline and flavonoid (Cluster 2 and Cluster 4). Hence, Sudanese and Sudano-Guinean honeys are promising for potential therapeutic applications because of their high total phenolic and flavonoid contents.

## 4. Conclusions

Physicochemical characteristics as well as total phenolic and flavonoid contents of honeys obtained from the three phytogeographical zones of Benin were compared in this study. The physicochemical characteristics show that these honeys are in agreement with the requirements set by the European Community and Codex Alimentarius Standards. Sudanese and Sudano-Guinean honeys are richer in phenolic compounds and proline than Guinean honeys. These results prove that specific areas and flora have a high impact on honey compositions. Additionally, honeys from the Sudanese and Sudano-Guinean areas show very interesting properties toward potential therapeutic applications. Overall, the influence of each phytogeographical area on honey composition is well-known, as well as the richness in polyphenols of Beninese honeys.

## Figures and Tables

**Table 1:** Physicochemical properties and chemical composition of honeys.

		Sudano-Guinean		Sudanese		Guinean
		Zone SG1 (Bassila) (*n* = 10)	Zone SG2 (N'Dali) (*n* = 10)	Zone S1 (Tanguieta) (*n* = 10)	Zone S2 (Kandi) (*n* = 10)	Zone G (Zogbodomey) (*n* = 10)
Color (mm Pfund)	Min	110.78 ± 0.26	163.15 ± 0.26	105.03 ± 1.05	357.02 ± 0.79	177.26 ± 1.84
	Max	554.60 ± 0.26	752.36 ± 0.53	448.75 ± 0.26	679.94 ± 1.05	333.43 ± 0.53
	Mean	295.98 ± 170.34^c^	414.04 ± 175.71^d^	265.39 ± 140.73^b^	534.76 ± 127.81^e^	250.84 ± 56.25^a^

Moisture (%)	Min	15.5 ± 0.00	17.5 ± 0.00	16.5 ± 0.00	16.75 ± 0.00	20.00 ± 0.00
	Max	21.95 ± 0.00	22.45 ± 0.07	21 ± 0.00	20 ± 0.00	23.50 ± 0.00
	Mean	18.75 ± 2.21^b^	19.17 ± 1.44^d^	18.9 ± 1.39^c^	17.64 ± 1.03^a^	21.06 ± 1.14^e^

pH	Min	5.00 ± 0.00	4.35 ± 0.07	4.10 ± 0.00	4.30 ± 0.00	2.87 ± 0.01
	Max	6.15 ± 0.07	4.75 ± 0.07	5.3 ± 0.00	4.80 ± 0.00	3.30 ± 0.02
	Mean	5.48 ± 0.43^d^	4.57 ± 0.14^c^	4.44 ± 0.36^b^	4.59 ± 0.15^c^	3.09 ± 0.14^a^

Acidity (meq/kg)	Min	9.00 ± 0.00	9.00 ± 0.00	17.75 ± 0.35	16.00 ± 0.00	10.00 ± 0.00
	Max	23.00 ± 0.00	30.00 ± 0.00	27.00 ± 0.00	18.75 ± 0.35	39.00 ± 0.00
	Mean	14.05 ± 4.23^a^	19.00 ± 5.25^c^	20.71 ± 3.01^d^	17.70 ± 0.88^b^	24.30 ± 7.18^e^

EC (mS/cm)	Min	0.68 ± 0.00	0.53 ± 0.00	0.42 ± 0.00	0.58 ± 0.00	0.37 ± 0.00
	Max	1.43 ± 0.00	1.07 ± 0.00	1.08 ± 0.00	0.84 ± 0.00	0.76 ± 0.01
	Mean	1.19 ± 0.23^d^	0.76 ± 0.20^c^	0.64 ± 0.22^b^	0.76 ± 0.09^c^	0.49 ± 0.14^a^

Fructose (g/100 g)	Min	21.67 ± 0.06	48.55 ± 0.04	29.07 ± 0.33	25.31 ± 0.10	43.82 ± 0.11
	Max	87.62 ± 0.06	74.23 ± 0.36	84.42 ± 0.00	94.21 ± 0.12	71.84 ± 0.24
	Mean	56.30 ± 19.30^c^	63.83 ± 9.07^e^	43.50 ± 17.30^a^	46.11 ± 20.13^b^	57.82 ± 7.63^d^

Proline (mg/kg)	Min	388.35 ± 0.93	506.63 ± 2.41	569.63 ± 0.90	698.10 ± 0.95	306.31 ± 0.84
	Max	894.16 ± 1.75	1187.93 ± 3.16	1055. 28 ± 1.70	1001.99 ± 1.94	894.72 ± 4.50
	Mean	618.41 ± 186.32^b^	836.54 ± 215.98^d^	824.76 ± 139.55^c^	845.30 ± 77.91^e^	464.36 ± 175.98^a^

Values with different letters on the same line are significantly different at 5%.

**Table 2:** Clustering of samples.

	Characteristics	Number of samples	Samples	Representative zones
Cluster 1	None	15	B1, B6, N5, N6, N9, T5, T6, T7, T9, T10, K2, K8, K9, K10, Z6	ZS: 50%
				ZSG: 25%
				ZG: 10%

Cluster 2	(i) Darkest	12	B2, B5, B9, N3, N4, N7, K1, K3, K4, K5, K6, K7	ZS: 30% ZSG: 30%
	(ii) High pH			
	(iii) High conductivity			
	(iv) High flavonoid content			

Cluster 3	(i) Low proline content	16	B3, B4, B7, B8, B10, N1, N10, T8, Z1, Z2, Z3, Z4, Z5, Z8, Z9, Z10	ZS: 5%
	(ii) Low phenolic content			ZSG: 35%
				ZG: 80%

Cluster 4	(i) High sugar content	2	N2, N8	ZSG: 10%
	(ii) High proline content			
	(iii) Low moisture			

Cluster 5	(i) Lightest	5	T1, T2, T3, T4, Z7	ZS: 20%
	(ii) High phenolic content			ZG: 10%

ZS: Sudanese zone; ZSG: Sudano-Guinean zone; ZG: Guinean zone. B: Bassila; N: N'Dali; K: Kandi; T: Tanguieta; Z: Zogbodomey.
